# Physician Burnout and Patient Safety Culture in Internal Medicine Departments: A Mixed-Methods Investigation Using an Investigator-Developed Questionnaire and Healthcare Provider Interviews

**DOI:** 10.7759/cureus.117678

**Published:** 2026-10-09

**Authors:** Harsha H Kumar, Chetan Rathi, Tanuja Manohar, Nawal Yoosaf

**Affiliations:** 1 Medicine, Jawaharlal Nehru Medical College, Belagavi, IND; 2 Internal Medicine, Sheikh Sultan Bin Zayed Hospital, Sharjah, ARE; 3 Medicine, N.K.P. Salve Institute of Medical Sciences and Research Centre and Lata Mangeshkar Hospital, Nagpur, IND; 4 Internal Medicine, Travancore Medicity Medical College Hospital, Kollam, IND

**Keywords:** emotional exhaustion, healthcare quality, internal medicine, mixed-methods study, patient safety culture, physician burnout

## Abstract

Background

Physician burnout is increasingly recognized as an important factor influencing healthcare quality, physician well-being, and patient safety culture in hospital settings. Internal medicine physicians may be particularly vulnerable to burnout due to high workload, extended duty hours, and complex clinical responsibilities. Using a mixed-methods approach, this study aimed to assess study-specific burnout classifications and perceived patient safety culture and to examine their association, while qualitatively exploring physicians' workplace experiences and perceived factors related to burnout and patient safety culture.

Methods

This cross-sectional mixed-methods study included 120 physicians working in the internal medicine department of a tertiary care hospital. Burnout and patient safety culture were assessed using an investigator-developed Physician Burnout and Patient Safety Culture Questionnaire, developed based on a review of published literature and domains commonly assessed by established instruments. Semi-structured interviews were conducted with a purposively selected subsample of 20 participants to explore their workplace experiences in greater depth. Quantitative data were analyzed using descriptive and inferential statistics, while qualitative data were analyzed using thematic analysis.

Results

A total of 120 physicians were included in the quantitative component, with 20 participants purposively selected for qualitative interviews. Overall, 86 (71.7%) participants were classified as having moderate or high burnout according to the study-specific scoring criteria, while high emotional exhaustion and depersonalization were each reported in 38 (31.7%) participants. A significant association was observed between study-specific burnout classification and perceived patient safety culture, with positive safety culture perceptions decreasing from 24 (70.6%) among participants with low burnout to 10 (25.0%) among those with high burnout (p < 0.001). Excessive workload, long duty hours, and poor work-life balance were frequently reported contributors to burnout, while 62 (51.7%) participants perceived an increased risk of medical errors. Qualitative findings further highlighted workload stress, staffing shortages, communication challenges, and fear of blame as important factors related to burnout and patient safety culture.

Conclusion

A substantial proportion of physicians were classified as having moderate or high burnout according to the study-specific scoring criteria, and higher burnout classifications were associated with less favorable perceptions of patient safety culture. Given the cross-sectional design and study-specific nature of the scoring system, these findings should not be interpreted as evidence of causality or as population prevalence estimates. Organizational strategies addressing workload, staffing, communication, and workplace culture may help support physician well-being and patient safety.

## Introduction

In recent years, the high rate of physician burnout has become an important occupational and public health issue within healthcare institutions, especially among physicians who work in internal medicine departments [1,2]. Burnout refers to the phenomenon of experiencing emotional exhaustion, depersonalization, and diminished personal achievement, which is caused by prolonged periods of job stress [1]. Physicians who practice in the field of internal medicine may be particularly vulnerable to burnout due to the number of hours they work, the number of patients they see, the amount of administrative duties they have, and the mental complexity involved in caring for patients with numerous injuries and illnesses [3,4]. Previous research has demonstrated that burnout negatively impacts the well-being of physicians, job satisfaction, and retention of doctors in the workforce [4].

Physician burnout impacts not only the physician but also the quality of care that the patient receives as well as the overall quality of the health care delivered. Studies have linked physician burnout to poor communication, lack of empathy, decreased attention to clinical decisions, and increased chances of physician errors [5,6]. These variables may create adherence issues to patient safety practices and result in negative health outcomes. In light of the heightened stress and growing emphasis on a people-oriented approach to care within healthcare institutions, it is important to evaluate the factors that influence the development of a patient safety culture in healthcare organizations [7].

The culture of patient safety is seen as the share value, belief, and behavior in the organization that motivates or obstructs the way in which safety practices and attitudes towards patient safety are adopted [8]. Creation of a culture of patient safety calls for teamwork, open communication, and a culture of accountability [7,8]. A weak patient safety culture may contribute to the underreporting of medical errors, poor communication, and preventable adverse events. Given these challenges, internal medicine departments are particularly demanding environments in which to practice medicine because of their complex use of multiple disciplines to care for patients, rapid patient turnover, and high clinical workload [9].

Recent research has demonstrated a strong association between physician burnout and patient safety cultur [10,11]. High levels of physician burnout may negatively affect physician involvement in behaviors that promote patient safety, while an unsupportive work environment and lack of organizational support may further contribute to physician burnout and adversely affect their workplace experience [10,12]. In spite of the numerous quantitative studies looking at physician burnout and the patient safety culture separately, fewer studies have combined both quantitative and qualitative approaches to assess physicians' experiences and attitudes towards workplace stress and patient safety practices.

Combining a mixed-methods research design may better demonstrate the impact of physician burnout and the patient safety culture by incorporating the use of previously validated quantitative measures of data collection, as well as descriptive analysis of the qualitative experiences of physicians in the workplace [7,11].

Thus, this mixed-methods study aimed to quantitatively assess physician burnout and perceived patient safety culture among physicians working in internal medicine departments and examine the association between these constructs and to qualitatively explore physicians’ workplace experiences and perceived factors related to burnout and patient safety culture.

## Materials and methods

Study design and setting

The study was conducted as a cross-sectional mixed-methods study in the Department of Medicine at N.K.P. Salve Institute of Medical Sciences and Research Centre and Lata Mangeshkar Hospital, Nagpur, Maharashtra, India. The study aimed to assess burnout and perceived patient safety culture using quantitative questionnaire-based assessment and qualitative semi-structured interviews. The study was conducted over a three-month period from February 2026 to April 2026 following approval from the Institutional Ethics Committee (NKPSIMS & RC and LMH/IEC/3/2026). The investigator-designed Physician Burnout and Patient Safety Culture Questionnaire was included in the study protocol and reviewed and approved by the Institutional Ethics Committee. Written informed consent was obtained from all participants, and confidentiality of the collected information was maintained.

Selection Criteria

Participants meeting the predefined eligibility criteria were recruited from the Department of Medicine during the study period. Eligible participants were physicians directly involved in patient care, including consultants and senior and junior residents, with at least six months of clinical experience in the Department of Internal Medicine and who provided informed consent.

Exclusion criteria included healthcare workers who were not directly involved in patient care, healthcare providers who were on long-term medical leave, temporary or visiting faculty members, those who had been associated with the Department of Internal Medicine for less than six months, and participants who failed to complete either of the study questionnaires.

Sample Size Calculation

The calculations for sample size determination for the quantitative part of the study were done using the single population proportion formula, assuming a burnout prevalence of 50%, with a level of confidence of 95% (Z = 1.96) and an allowable error of 9% using the formula n = Z² × p × (1 − p) / d², where n refers to the sample size, Z corresponds to the level of confidence of 95%, p stands for the estimated prevalence, and d means allowable error. Taking into account the sampling nonresponse rate, the final sample size for the quantitative part of the study was equal to 120 respondents. In the qualitative part of the study, 20 respondents were selected purposively to ensure variability in burnout experience, level of profession, and work experience.

Data sources and variables

Development of the Physician Burnout and Patient Safety Culture Questionnaire

Quantitative data were collected using a Physician Burnout and Patient Safety Culture Questionnaire developed by the investigators following a review of relevant published literature and established tools, including the Maslach Burnout Inventory-Human Services Survey (MBI-HSS) and Hospital Survey on Patient Safety Culture (HSOPSC) [1,7]. The MBI-HSS and HSOPSC were used only as conceptual references for identifying relevant domains and were not administered, combined, or modified for use in this study. The investigator-developed questionnaire assessed burnout and patient safety culture domains, along with demographic characteristics. The questionnaire was reviewed and approved by the Institutional Ethics Committee before study commencement. Qualitative data were collected through semi-structured interviews exploring workload, emotional pressure, communication issues, and investigative practices.

Questionnaire Scoring

The final investigator-developed questionnaire consisted of 23 items covering physician burnout, professional well-being, workload-related stress, patient safety culture, communication, teamwork, organizational support, and safety-related perceptions. Each item was rated on a five-point Likert scale ranging from 1 (strongly disagree) to 5 (strongly agree). Content validity was assessed before study commencement by a panel of five experts comprising two consultants in internal medicine, one psychiatrist/clinical psychologist, one healthcare quality and patient-safety expert, and one public health/medical education expert. Each item was independently rated for relevance using a four-point scale ranging from 1 (not relevant) to 4 (highly relevant). The item-level Content Validity Index (I-CVI) was calculated as the proportion of experts assigning a rating of 3 or 4 to each item. All 23 items received a relevance rating of 3 or 4 from all five experts, resulting in an I-CVI of 1.00 for each item. The scale-level Content Validity Index was calculated as the average of the I-CVI values (S-CVI/Ave), which was 1.00. Internal consistency was assessed using Cronbach's alpha, which was 0.865. Given the multidomain nature of the questionnaire, Cronbach's alpha was interpreted as an indicator of overall internal consistency and not as evidence of construct validity. The questionnaire was reviewed and approved by the Institutional Ethics Committee before study commencement.

Positively worded items were scored directly, whereas negatively worded items (items 1, 2, 3, 4, 6, 7, 16, 17, 21, and 23) were reverse-scored. Item scores were summed to obtain a total score ranging from 23 to 115. Higher total scores indicated more favorable physician well-being, stronger perceptions of patient safety culture, greater organizational support, and lower levels of burnout. For descriptive analysis, total scores were classified according to the predefined categories described in Appendix A. For study-specific interpretation, total scores < 58 were categorized as poor physician well-being and patient safety culture, scores of 58-86 as moderate, and scores > 86 as favorable. For burnout classification, scores < 58 were categorized as high burnout, scores of 58-86 as moderate burnout, and scores > 86 as low burnout. All 23 questionnaire items contributed to the total score. Because the questionnaire incorporated items from several related domains, the overall score reflects a composite assessment of physician well-being and patient safety-related perceptions rather than a formally validated measure of a single construct. These thresholds were used solely for descriptive classification within the present study and should not be interpreted as validated diagnostic or population prevalence thresholds.

Patient Safety Culture Classification

For the categorical analysis, patient safety culture was assessed using the 10 questionnaire items mapped to the patient safety culture domain (items 9, 10, 11, 12, 13, 15, 18, 19, 22, and 23). Each item was scored from 1 to 5 after reverse scoring of negatively worded item 23. The resulting patient safety culture domain score ranged from 10 to 50, with higher scores indicating more favorable perceptions of patient safety culture. A score corresponding to at least 70% of the maximum attainable domain score was predefined as a positive perception of patient safety culture. Accordingly, scores of ≥35 were classified as positive, whereas scores of <35 were classified as negative. This binary classification was used for the secondary categorical analysis examining the association between study-specific burnout classification and perceived patient safety culture.

Item-to-Domain Mapping

The questionnaire items were mapped to their principal conceptual domains (Table 1). This allocation was based on the content of individual items and was intended to clarify the constructs represented in the instrument rather than to establish formally validated subscales.

**Table 1:** Item-to-domain allocation of the investigator-developed questionnaire

Domain	Items
Burnout-related experiences	1, 2, 3, 4, 6, 7, 21
Professional well-being/achievement	5, 20
Patient safety culture	9, 10, 11, 12, 13, 15, 18, 19, 22, 23
Organizational support	14
Workload/administrative burden	6, 7, 16, 21
Perceived impact of burnout on patient safety	17

Data Collection

Qualitative data were collected through paper questionnaires and semi-structured interviews conducted by the researchers. Each participant was assigned a unique study identification number to ensure confidentiality. The completed questionnaires and consent forms were stored securely and were accessible only to the research team. Electronic data were stored in a password-protected system accessible only to authorized members of the research team. The interview guide was developed in accordance with the study objectives and addressed workload-related stress, emotional pressure, communication issues, organizational challenges, patient safety practices, coping mechanisms, and perceived effects of burnout on patient safety.

Statistical analysis

Quantitative data were entered into Microsoft Excel (Microsoft Corporation, Redmond, WA) and analyzed using the Statistical Package for the Social Sciences (SPSS) version 27.0 (IBM Corp., Armonk, NY, USA). Continuous variables are presented as mean ± standard deviation (SD), whereas categorical variables are expressed as frequencies and percentages. The Pearson chi-square test was used to assess the association between study-specific burnout classification and the binary patient safety culture classification. Cramer's V was calculated to quantify the strength of the association. A two-tailed p-value of <0.05 was considered statistically significant. The categorical association between burnout classification and perceived patient safety culture was considered a secondary analysis. Only completed questionnaires were included in the quantitative analysis; no imputation of missing questionnaire responses was performed.

Qualitative data were analyzed using thematic analysis. Interviews were audio-recorded with participants' consent and transcribed verbatim. Initial codes were identified from the transcripts and organized into broader categories and themes, including workplace stressors, organizational challenges, communication barriers, coping strategies, and the perceived impact of burnout on patient safety. Interviews continued until thematic saturation was reached, defined as the point at which successive interviews generated no substantially new ideas or themes relevant to the study objectives.

A mixed-methods approach was used, with quantitative and qualitative components conducted within the same study period. Quantitative findings provided descriptive information on burnout and perceived patient safety culture, while qualitative interviews explored participants' workplace experiences and perceived contributors to burnout and patient safety concerns. Findings from the two components were integrated during interpretation to provide contextual understanding of the quantitative findings.

## Results

Table 2 presents demographic and professional characteristics of study participants. More than half of the participants reported working over 60 hours a week, and many had more than five years of clinical experience.

**Table 2:** Demographic and professional characteristics of study participants (N=120) Values are presented as mean ± SD or n (%) SD, standard deviation

Parameter	Value
Mean age (years)	34.8 ± 6.9
Gender	
Male	78 (65.0%)
Female	42 (35.0%)
Designation	
Junior residents	48 (40.0%)
Senior residents	44 (36.7%)
Consultants	28 (23.3%)
Clinical experience >5 years	52 (43.3%)
Weekly working hours >60	66 (55.0%)

Burnout classifications and burnout-related dimensions for respondents, as determined using the study-specific scoring criteria, are presented in Table 3. Overall, 86 (71.7%) participants were classified as having moderate or high burnout according to these criteria. High emotional exhaustion and depersonalization were each reported in 38 (31.7%) participants.

**Table 3:** Burnout levels and burnout dimensions distribution among participants (N=120) Values are presented as n (%).

Parameter	Frequency (%)
Overall burnout level	
Low	34 (28.3%)
Moderate	46 (38.3%)
High	40 (33.3%)
Burnout dimensions	
High emotional exhaustion	38 (31.7%)
High depersonalization	38 (31.7%)
Low personal accomplishment	36 (30.0%)

Table 4 presents mean scores for patient safety culture domains; teamwork and communication openness had higher mean scores, whereas staffing and non-punitive response to errors had the lowest scores.

**Table 4:** Patient safety culture domain scores among participants Values are presented as mean ± SD SD, standard deviation

Patient safety culture domain	Mean score ± SD
Teamwork	3.8 ± 0.6
Communication openness	3.5 ± 0.7
Frequency of events reported	3.2 ± 0.7
Staffing	2.9 ± 0.8
Non-punitive response to errors	2.7 ± 0.6

Table 5 presents the association between study-specific burnout categories and perceived patient safety culture.

**Table 5:** Association between study-specific burnout classification and perceived patient safety culture Values are presented as frequency (percentage). Patient safety culture was assessed using items 9, 10, 11, 12, 13, 15, 18, 19, 22, and 23. The resulting score ranged from 10 to 50; scores ≥ 35 were classified as positive and scores < 35 as negative, based on the predefined criterion of ≥70% of the maximum attainable score. Pearson's chi-square test was used to assess the association between study-specific burnout classification and perceived patient safety culture. Cramer's V was 0.36, indicating a moderate association. A two-sided p-value of <0.05 was considered statistically significant.

Burnout level	Positive safety culture, n (%)	Negative safety culture, n (%)	χ²	p-value
Low burnout (n = 34)	24 (70.6)	10 (29.4)	15.39	<0.001
Moderate burnout (n = 46)	22 (47.8)	24 (52.2)		
High burnout (n = 40)	10 (25.0)	30 (75.0)		

Pearson's chi-square test demonstrated a significant association between study-specific burnout classification and perceived patient safety culture (χ² = 15.39, p < 0.001). The strength of the association was moderate (Cramer's V = 0.36). The proportion of participants classified as having a positive perception of patient safety culture decreased progressively across the low, moderate, and high burnout categories, from 70.6% to 47.8% and 25.0%, respectively. These findings represent an association within the study-specific scoring framework and should not be interpreted as evidence of a causal relationship or as an independently validated relationship between burnout and patient safety culture.

Table 6 summarizes work-related contributors to burnout and participants’ perceptions of its impact on patient safety. Excessive workload, long duty hours, and poor work-life balance were the most commonly reported contributors. More than half of the participants perceived that burnout increased the risk of medical errors.

**Table 6:** Work-related contributors to burnout and perceived impact on patient safety (N=120) Values are presented as n (%).

Parameter	Frequency (%)
Contributors to burnout	
Excessive workload	78 (65.0%)
Long duty hours	72 (60.0%)
Poor work-life balance	70 (58.3%)
Administrative burden	64 (53.3%)
Inadequate staffing	58 (48.3%)
Perceived impact on patient safety	
Increased risk of medical errors	62 (51.7%)
Reduced communication quality	54 (45.0%)
Delayed decision-making	46 (38.3%)

Participants have identified workload-related stress, communication barriers, staffing shortages, and fear of blame as key contributors to their experiencing burnout, as well as major challenges to maintaining patient safety culture throughout the qualitative interviews that have taken place. Table 7 highlights these main themes from the qualitative interviews.

**Table 7:** Qualitative themes identified during interviews

Theme	Key findings
Workload stress	Long duty hours and high patient load
Organizational challenges	Staffing shortages and administrative burden
Communication barriers	Hierarchical communication and fear of blame
Emotional impact	Fatigue, reduced motivation, emotional exhaustion
Coping mechanisms	Peer support and personal resilience

These results suggest that workload-related stress, communication barriers, staffing shortages, and fear of blame are key contributors to burnout and challenges in maintaining a positive patient safety culture.

## Discussion

This mixed-methods study assessed study-specific burnout classifications and perceived patient safety culture among healthcare providers working in an internal medicine department. Additionally, contributing factors to burnout and patient safety concerns were explored through workplace experiences. Results indicated that a substantial proportion of participants were classified as having moderate or high burnout according to the study-specific scoring criteria. Higher burnout classification was also associated with less favorable perceptions of patient safety culture, indicating an observed relationship between physician well-being and perceptions of organizational safety practices. Burnout and patient safety culture were assessed using an investigator-developed questionnaire informed by published burnout and patient safety culture literature.

Compared with previous literature [10,11], 86 (71.7%) participants in the present study were classified as having moderate or high burnout according to the study-specific scoring criteria. These findings should be interpreted as descriptive classifications rather than estimates of burnout prevalence based on a validated instrument. Internal medicine departments frequently involve long working hours, high patient volumes, complex clinical decision-making, and substantial administrative responsibilities. Each of these factors may place healthcare providers at increased risk of chronic work stress and emotional fatigue [3,12]. The present findings therefore highlight a substantial burden of burnout-related symptoms within the study population.

Emotional exhaustion was the most frequently reported high-level burnout dimension among the participants. Previous research has also indicated that excessive workload, extended duty hours, and inadequate time for recovery from work can adversely affect a physician's level of emotional exhaustion [13]. Mental fatigue, lack of motivation or energy, and difficulty maintaining an appropriate work-life balance were frequently identified during the qualitative interviews. These findings were consistent with the quantitative assessment and provided additional context regarding the severity of burnout-related experiences among participants. Findings from this study demonstrated an association between study-specific burnout classification and perceived patient safety culture, with a lower proportion of participants reporting positive safety culture perceptions across increasing burnout categories.

The observed association between burnout classification and perceived patient safety culture should be interpreted cautiously because both domains were assessed using the same investigator-developed questionnaire. Consequently, some degree of measurement overlap between the constructs cannot be excluded. The findings therefore indicate an observed association within the present study rather than evidence of an independently validated relationship between two separately validated constructs.

Participants with higher burnout classifications had lower proportions of positive perceptions of teamwork, communication openness, staffing, and non-punitive responses to errors than participants with lower burnout classifications. The existing literature suggests that physician burnout may adversely affect communication, clinical attentiveness, teamwork, and adherence to patient safety practices [5,14]. Physicians experiencing high levels of emotional stress and fatigue may also experience impaired cognitive performance and reduced engagement in safety-promoting behaviors.

Inadequate staffing and organizational stressors are important contributors to burnout and are also relevant to concerns about patient safety. Participants in this study identified excessive workload, administrative burden, and inadequate staffing as barriers to providing high-quality patient care. Similar findings have been reported in previous studies examining the healthcare workplace environment and physician well-being [15,16]. Insufficient staffing may increase workload, contribute to delayed decision-making and miscommunication, and increase concerns regarding patient safety.

Qualitative results provided additional insight into physicians' experiences and perceptions of patient safety culture. During interviews, participants identified fear of blame for errors, hierarchical communication patterns, and lack of organizational support as barriers to developing a positive patient safety culture. These findings are consistent with previous literature emphasizing the importance of open communication, supportive leadership, and non-punitive reporting systems in developing a positive patient safety culture [8,17]. Workplaces that do not promote open dialogue about errors may contribute to emotional stress and reduce the likelihood of reporting safety-related incidents.

The integration of quantitative burnout and patient safety culture scores with qualitative experiential data enabled triangulation of findings and provided a more comprehensive understanding of factors influencing physician burnout and patient safety culture. The use of mixed methods therefore allowed for a more comprehensive evaluation of the relationship between physician burnout and organizational safety practices than the use of either method alone [7,11]. The findings from this study highlight potential areas for institutional strategies aimed at reducing burnout-related symptoms and strengthening patient safety culture. Institution-wide strategies that target workload optimization, provide staffing support, develop wellness programs for physicians, provide communication skills training, and promote supportive leadership may enhance physician well-being and contribute to safer environments for patient care [18].

Strengths and limitations

There are several strengths to this study. The integrated mixed-methods approach combined quantitative questionnaire-based assessment with qualitative interviews, allowing a broader evaluation of physician burnout and perceived patient safety culture within the same population. The study also provides a transparent description of the investigator-developed questionnaire, including its content-validation process, item-to-domain mapping, scoring procedures, and internal consistency assessment.

The study was conducted at a single institution with a relatively small sample, which may limit the generalizability of the findings. Self-reported questionnaire responses and interviews may have introduced social desirability and recall biases. Although the questionnaire demonstrated strong content validity and acceptable internal consistency, formal construct and criterion validity were not established, and the scoring thresholds were developed for study-specific classification rather than validated diagnostic assessment; therefore, burnout classifications should not be interpreted as population prevalence estimates or as equivalent to classifications obtained using validated burnout instruments. The patient safety culture classification was also based on a predefined threshold within the investigator-developed instrument and should be interpreted within the context of the study-specific scoring framework. The response rate and potential selection bias could not be fully assessed because detailed records of the number of eligible physicians approached were unavailable. Purposive sampling of the qualitative component may not capture the full range of experiences across different professional or institutional settings. Finally, the cross-sectional design precludes establishing temporal or causal relationships between physician burnout and perceived patient safety culture.

## Conclusions

Physicians working in an internal medicine department demonstrated a substantial burden of burnout-related symptoms and suboptimal perceptions of patient safety culture, with a significant association observed between study-specific burnout classifications and perceived patient safety culture. Emotional exhaustion, high workload, inadequate staffing, and organizational stressors were important factors identified in relation to burnout and workplace safety concerns. The mixed-methods approach provided quantitative assessment of burnout and patient safety perceptions alongside qualitative insights into physicians' workplace experiences. Given the study-specific nature of the questionnaire and its scoring thresholds, the findings should be interpreted cautiously and should not be considered estimates of population burnout prevalence. Future studies using independently validated instruments and prospective or longitudinal designs are warranted to further evaluate the relationship between physician burnout and patient safety culture and to inform organizational strategies aimed at improving physician well-being, communication, staffing, and patient safety.

## Appendices

Appendix A: Investigator-developed physician burnout and patient safety culture questionnaire

Development of the Questionnaire

The physician burnout and patient safety culture questionnaire was developed by the investigators following a review of published literature on physician burnout and patient safety culture, including domains commonly assessed in established instruments such as the Maslach Burnout Inventory-Human Services Survey (MBI-HSS) and the Hospital Survey on Patient Safety Culture (HSOPSC) [1,7]. The questionnaire was reviewed and approved as part of the Institutional Ethics Committee-approved study protocol and was used solely for research purposes within the present study.

Instructions

Participants were asked to indicate the response that best reflected their experiences and perceptions during the preceding three months. Response scale: 1 = strongly disagree, 2 = disagree, 3 = neutral, 4 = agree, 5 = strongly agree.

**Table 8:** Investigator-developed physician burnout and patient safety culture questionnaire Response scale: 1 = strongly disagree, 2 = disagree, 3 = neutral, 4 = agree, and 5 = strongly agree.

Item No.	Question
1	I feel emotionally exhausted because of my work responsibilities.
2	I feel physically drained at the end of a typical working day.
3	I find it difficult to maintain a healthy work-life balance.
4	I feel detached or emotionally distant from some patients because of work-related stress.
5	I feel that my work positively contributes to patient care and professional achievement.
6	Excessive workload negatively affects my ability to perform my duties effectively.
7	Long working hours contribute to stress and fatigue in my professional life.
8	Staffing levels in my department are adequate to support safe patient care.
9	Teamwork among healthcare professionals in my department is effective.
10	Communication among members of the healthcare team is open and constructive.
11	I feel comfortable reporting patient safety concerns or medical errors.
12	Staff members are treated fairly when errors or adverse events occur.
13	My department promotes a culture that prioritizes patient safety.
14	Organizational support is available when physicians experience work-related stress or burnout.
15	Patient safety incidents are discussed openly to improve future practice.
16	Administrative duties interfere with my ability to provide direct patient care.
17	Burnout among healthcare professionals may increase the risk of medical errors.
18	I am satisfied with the overall safety culture within my department.
19	I feel confident that patient safety is consistently maintained despite workload pressures.
20	Overall, I am satisfied with my professional well-being in my current work environment.
21	High patient volume contributes significantly to my work-related stress.
22	I regularly report patient safety incidents, near misses, or safety concerns when they occur.
23	Fear of blame discourages healthcare professionals from reporting errors or patient safety concerns.

Scoring and Interpretation

Each item was scored on a five-point Likert scale ranging from 1 to 5. For positively worded items, higher scores reflected more favorable perceptions of professional well-being and patient safety culture. Negatively worded items (items 1, 2, 3, 4, 6, 7, 16, 17, 21, and 23) were reverse-scored before calculation of total scores.

Individual item scores were summed to generate a total score ranging from 23 to 115. Interpretation of total scores: scores <58 indicated poor physician well-being and patient safety culture; scores of 58-86 indicated moderate physician well-being and patient safety culture; and scores >86 indicated favorable physician well-being and patient safety culture. Higher total scores indicated lower levels of burnout, stronger perceptions of patient safety culture, greater organizational support, more positive workplace experiences, and greater confidence in patient safety practices among physicians.
